# Impact of Facial Conformation on Canine Health: Corneal Ulceration

**DOI:** 10.1371/journal.pone.0123827

**Published:** 2015-05-13

**Authors:** Rowena M. A. Packer, Anke Hendricks, Charlotte C. Burn

**Affiliations:** 1 Department of Clinical Science and Services, Royal Veterinary College, University of London, Hertfordshire, AL9 7TA, United Kingdom; 2 Department of Production and Population Health, Royal Veterinary College, University of London, Hertfordshire, AL9 7TA, United Kingdom; Faculty of Animal Sciences and Food Engineering, University of São Paulo, BRAZIL

## Abstract

Concern has arisen in recent years that selection for extreme facial morphology in the domestic dog may be leading to an increased frequency of eye disorders. Corneal ulcers are a common and painful eye problem in domestic dogs that can lead to scarring and/or perforation of the cornea, potentially causing blindness. Exaggerated juvenile-like craniofacial conformations and wide eyes have been suspected as risk factors for corneal ulceration. This study aimed to quantify the relationship between corneal ulceration risk and conformational factors including relative eyelid aperture width, brachycephalic (short-muzzled) skull shape, the presence of a nasal fold (wrinkle), and exposed eye-white. A 14 month cross-sectional study of dogs entering a large UK based small animal referral hospital for both corneal ulcers and unrelated disorders was carried out. Dogs were classed as affected if they were diagnosed with a corneal ulcer using fluorescein dye while at the hospital (whether referred for this disorder or not), or if a previous diagnosis of corneal ulcer(s) was documented in the dogs’ histories. Of 700 dogs recruited, measured and clinically examined, 31 were affected by corneal ulcers. Most cases were male (71%), small breed dogs (mean± SE weight: 11.4±1.1 kg), with the most commonly diagnosed breed being the Pug. Dogs with nasal folds were nearly five times more likely to be affected by corneal ulcers than those without, and brachycephalic dogs (craniofacial ratio <0.5) were twenty times more likely to be affected than non-brachycephalic dogs. A 10% increase in relative eyelid aperture width more than tripled the ulcer risk. Exposed eye-white was associated with a nearly three times increased risk. The results demonstrate that artificially selecting for these facial characteristics greatly heightens the risk of corneal ulcers, and such selection should thus be discouraged to improve canine welfare.

## Introduction

Concern has arisen in recent years that artificial selection for extreme facial morphologies in the domestic dog may be leading to an increased frequency of eye disorders [1,2]. It has previously been identified that there is an average of 6.6 different eye disorders that can affect each breed (of the 148 examined); however, in some breeds there were many more, with the American Cocker Spaniel, Miniature and Toy Poodle and Pekingese reported to have 18 or more disorders [3]. Corneal ulceration is one of the most common eye diseases in domestic dogs [4,5], and is a major cause of blindness [6]. It is presumed that damage to the cornea can cause substantial pain as the cornea is densely innervated by nociceptive afferent axons [7]. Corneal ulcers may result from varied aetiologies including trauma, preocular tear film defects or deficiencies; irritants, eyelid or eyelash abnormalities; immune-mediated or allergic inflammation; foreign bodies or the inability to blink [8,9]. In addition, breed predispositions due to craniofacial and eyelid conformation have been implicated as risk factors for corneal ulcers [10]. Some ‘breed standards’, the official description of how each breed should ideally look, advocate extreme eye morphologies, and have been implicated as a potential driver of this problem [11].

### Breed standards and ocular health

Some members of the veterinary community have stated their objection to the conformational features written into breed standards which potentially jeopardise the health of the eye [12]. Stades *et al*. (2007) stated that the short muzzle, nasal fold, profuse hair and large, protruding eyes of the Pekingese were likely to cause problems including: nasal fold trichiasis (rubbing of the nasal fold against the surface of the eye); medial entropion (in-rolling of the inner corner of the eyelid, rubbing against the surface of the eye); macropalpebral fissures (over-large eyelid openings); and lagophthalamos (inability to blink fully). Consequently, Stades and colleagues stated that “*most veterinarians do not agree with [these breed standards]”*[13]. Characteristically round, prominent eyes, have previously been described by breed standards internationally, e.g. for the US Pug standard it is stated that, “*the eyes are dark in color*, *very large*, *bold and prominent*, *globular in shape*” [14]. There are concerns regarding potential pathological implications of this conformation, and it has been stated that “*because of breed standards and fashions that disregard the animals’ health but are nevertheless supported by breeders*, *judges*, *and buyers alike*, *almost all eyes of prominent-eyed breeds are chronically irritated and predisposed to luxation*” [13].

Following recent pressures from the general public, animal welfare charities and the veterinary community, changes were made to breed standards in the UK to discourage some of the extreme morphologies described above (Table 1). Such changes have not been widely implemented in the US, with explicit examples of the encouragement of ‘very large’ eyes still present in American Kennel Club breed standards (Table 1).

**Table 1:** Kennel Club and American Kennel Club breed standard references to the visible size of the eye.

Breed	Kennel Club	American Kennel Club
Griffon Bruxellois	Black-rimmed, very dark, [delete ‘large’] round, clear, alert and not too large. [44]	Eyes set well apart, very large, black, prominent, and well open.
Japanese Chin	Moderately large, dark, set far apart. Size should be in proportion to size of skull. [Delete ‘Most desirable that’] Small amount of white shows in the inner corners, giving characteristic look of astonishment. [Delete ‘(wrongly called squint) which should on no account be lost’.] Eyes should be forward facing, not set on side of head. [45]	Set wide apart, large, round, dark in color, and lustrous. A small amount of white showing in the inner corners of the eyes is a breed characteristic that gives the dog a look of astonishment.
King Charles Spaniel (English Toy Spaniel)	Relatively [delete ‘very’] large, dark, set wide apart, eyelids block square to face line, pleasing expression. [46]	Large and very dark brown or black, set squarely on line with the nose, with little or no white showing.
Pug	Dark, [delete ‘very’] not too large, round [delete ‘globular’] in shape, soft and solicitous in expression, very lustrous, and when excited, full of fire. Never protruding, exaggerated or showing white. Free from obvious eye problems. [47]	The eyes are dark in color, very large, bold and prominent, globular in shape, soft and solicitous in expression, very lustrous, and, when excited, full of fire.
Boston Terrier	Wide apart, round and not too large dark in colour; expression alert, kind and intelligent. [48]	The eyes are wide apart, large and round and dark in color.
Pekingese	Clear, round, dark, lustrous and not too large. Free from obvious eye problems. [49]	They are large, very dark, round, lustrous and set wide apart. The look is bold, not bulging. The eye rims are black and the white of the eye does not show when the dog is looking straight ahead.

(UK) Kennel Club breed standards include the recent amendments to avoid exaggerated morphologies, denoted by underlining additions, and square brackets and inverted commas notifying deletions. N.B. Although ‘relatively’ or ‘not too large’ were added as modifiers to ‘large eyes’ in these examples, other breed standards still consider ‘large’ eyes as a desirable feature, including the Shih Tzu [42] and the Cavalier King Charles Spaniel [43] standards.

Recommendations from the Independent Inquiry into Dog Breeding (‘The Bateson Inquiry’ [15]) included the suggestion that extreme morphologies that can damage or have been demonstrated to directly threaten health and welfare should be avoided. Furthermore, the Inquiry suggested that where welfare problems exist in a breed, breed standards should be amended specifically to encourage the selection of morphologies that will improve welfare. The proposed changes to standards were to include diagrams or quantitative ratios, to be both more precise and in order to encourage the necessary changes. Prior to the current study there were no data available quantitatively linking craniofacial conformational traits with ophthalmic disease, and thus it is not known whether the changes that have been made thus far (in the UK) are sufficient to protect ophthalmic health. It is not yet known which precise conformational traits should be ‘avoided’, as suggested by Bateson [15]. This study aims to investigate the potential relationship between craniofacial conformation and corneal ulceration, which may aid further breed standard revisions internationally and may aid breeders in avoiding high-risk morphologies.

### Craniofacial risk factors for corneal ulcers

Several conformational features of the domestic dog, observed in several popular companion dog breeds, have been anecdotally implicated as increasing corneal ulcer risk. Here we focus on those often seen in brachycephalic (short-muzzled) dogs.

#### Prominent eyes

Although there is relatively little variation in globe size between dog breeds [16], comparatively large palpebral fissures (eyelid apertures) are frequently observed in brachycephalic dogs. In these dogs, large palpebral fissures are frequently accompanied by shallow orbits, leading to abnormally protruding eyes [17] at risk of trauma from external insults. The prominent eye conformation can lead to a physical inability to close the eyelids completely [17,18]. The failure to blink adequately (lagophthalmos) compromises the spreading of the protective tear film, leading to areas of corneal drying with secondary erosion and ulceration [6,19]. Surgical intervention (medial canthoplasty) is often carried out to shorten the large palpebral fissure by 6–8mm, thus reducing lagophthalmic complications along with the risk of globe prolapse (dislocation of the eyeball), a further problem observed in breeds with large, prominent eyes [13].

#### Visible sclera

The prominent eye of some dogs is associated with scleral exposure (the white part of the eye, termed “eye white” or “white of eye” in some breed standards). Because globe size is so consistent across dog breeds [16], it follows that visibility of the white part of the eye may be due to extra-large palpebral fissures and/or extremely shallow orbits. However, visibility of the white part of the eye as a predictor of eye disorders has not been quantitatively investigated previously. In the Japanese Chin breed standard ‘eye white in the inner corners’ (nasal sclera exposure) is encouraged (Table 1), whereas in other standards it is discouraged.

#### Nasal folds

Another potentially relevant conformational feature commonly seen in brachycephalic dogs is a nasal fold, also known as an ‘over nose wrinkle’ or ‘nose rope’ in the dog showing community. This feature is found in several brachycephalic breeds where the skin overlying the short muzzle is not reduced in proportion to the facial skeleton, resulting in the excessive skin being forced into wrinkles. The fold, or hairs growing from the nasal fold, can rub against the cornea of the prominent eye causing painful traumatic keratitis and ulceration, termed ‘nasal fold trichiasis’. This contact can be continual or positional (e.g. only rubbing when the dog looks to one side) and is most common in the more extreme brachycephalic breeds [20]. Nasal fold trichiasis may require surgical resection of the fold [21] or in cases where a large palpebral fissure and nasal fold trichiasis are present, medial canthoplasty may help to eliminate the contact between the cornea and nasal fold [22].

Nasal folds are stated as a desirable feature in several brachycephalic breeds, as seen in the breed standards below (Table 2). Following the introduction of ‘Breed Watch’, a (UK) Kennel Club initiative to highlight potential exaggerations in individual breeds, the Bulldog [23], Pekingese [24] and Pug [25] have all had points of concern highlighted for special attention by judges that are related to nasal folds (Table 2). This feature is still allowed, although exaggerated versions of it are discouraged. Nasal folds are also encouraged in American Kennel Club breed standards, with descriptions of this feature including: “…*heavy wrinkles forming a soft roll over the extremely short nose*” for the French Bulldog [26].

**Table 2:** Nasal folds in breed standards and nasal fold related statements from The Kennel Club (UK) ‘Breed Watch’ initiative.

Breed	Breed standard text referring to the presence of a nasal fold	Kennel Club ‘Breed Watch’ points of concern for special attention by judges
Pekingese	A slight wrinkle, preferably broken, may extend from the cheeks to the bridge of the nose in a wide inverted ‘v’. This must never adversely affect or obscure eyes or nose. [49]	Heavy over nose wrinkle and prominent nasal folds
Bulldog	Over nose wrinkle, if present, whole or broken, must never adversely affect or obscure eyes or nose. Pinched nostrils and heavy over nose roll are unacceptable and should be heavily penalised. [50]	Heavy overnose wrinkle (roll); Excessive amounts of loose skin that impinge the eye (e.g. from nasal folds)
Pug	Wrinkles on forehead clearly defined without exaggeration. Eyes or nose never adversely affected or obscured by over nose wrinkle. [47]	Excessive nasal folds

### Aims

The aim of this study was to confirm the extent to which the aforementioned common conformational features of the domestic dog—wide palpebral fissures, nasal folds, the brachycephalic craniofacial conformation and the presence of visible sclera—do or do not increase the risk of corneal ulcers.

## Methods

### Recruitment of owners and study dogs

Between December 2010 and January 2012, dogs referred to the Royal Veterinary College (RVC) Small Animal Referral Hospital (SARH) were recruited for inclusion in the study. Owners of dogs referred to any clinical service for a routine appointment were approached. As appointments were booked in advance, all dogs were considered for recruitment prior to their arrival at the hospital and were excluded on a case-by-case basis if they were:

- Presented for a disorder that would make them unsuited to leaving wards/nursing care during their stay in the hospital, or too painful/uncomfortable to be handled.
- Known to be aggressive and therefore not suitable for handling
- Isolated from the general hospital population for infection control
- Already recruited to a separate clinical trial/study within the study hospital

The owners of the remaining dogs were approached in the waiting room before their consultation, to explain the purpose of the study and to request informed consent for their dog. This study was approved by the RVC’s Ethical Review Committee (reference number: URN 2010 1054).

### Clinical classification

**Fig 1:**
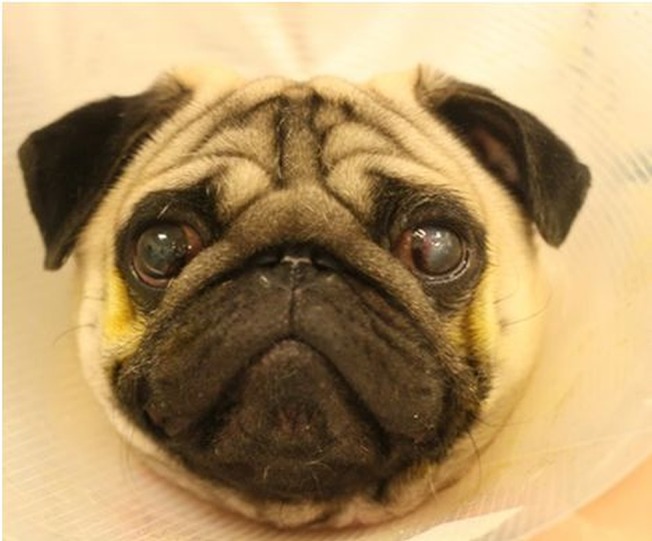
A 2 year old male Pug diagnosed and undergoing treatment for a corneal ulcer in his left eye.

Dogs were classed as affected if they were diagnosed with a corneal ulcer following the use of fluorescein dye while at the SARH (whether referred for this disorder or other unrelated disorders), or if a previous diagnosis of corneal ulcer(s) was documented in the dogs’ histories (Fig 1). As this study involved dogs affected by a wide variety of disorders, dogs diagnosed with corneal ulcers included both those referred to a single ophthalmology specialist specifically for this condition, and dogs referred to the SARH for other conditions, diagnosed with an ulcer by their attending clinician on other clinical services. All owners were asked whether their dog had a history of eye problems in a generic owner questionnaire (S1 Table), which aided in the identification of dogs previously treated for corneal ulcers. If insufficient information regarding historical ulcers was provided in the dog’s referral history, or owners were unsure of prior ophthalmic disorders in their dog, then the first opinion practice was contacted to provide this information and confirm a history of this condition.

#### Morphometrics

**Fig 2:**
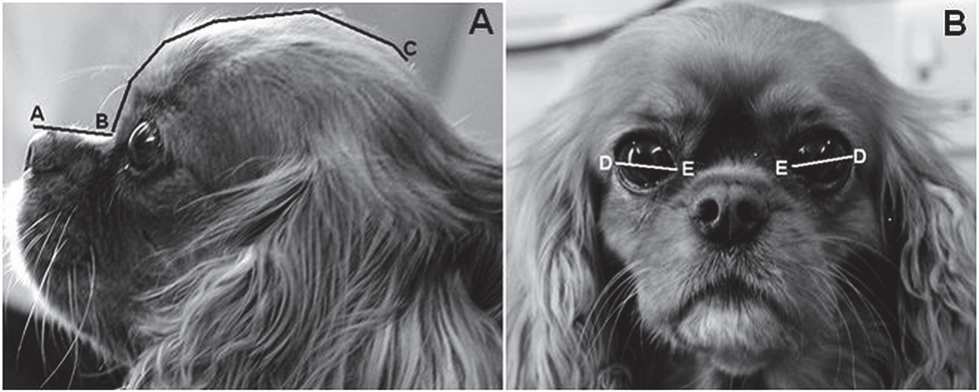
Quantifying muzzle length (A-B), cranial length (B-C) and palpebral fissure width (D-E). **A, left:** Muzzle length is defined as the distance (mm) from the dorsal tip of the nasal planum to the stop. Cranial length is defined as the distance (mm) from the stop to the occipital protruberance. **B, right:** Palpebral fissure width is defined as the straight-line distance (mm) between the medial and lateral canthus. As an example, this Cavalier King Charles Spaniel has a craniofacial ratio of 0.27 (muzzle length 28mm / cranial length 102mm), and a relative palpebral fissure width value of 33.3% ((palpebral fissure width 34mm / cranial length 102mm) *100)

Morphometric data were collected for each dog using previously defined measuring protocols [27], measuring 11 conformational features that were demonstrated to be breed-defining: muzzle length, cranial length, head width, eye width, neck length, neck girth, chest girth, chest width, body length, height at the withers and height at the base of tail (all in cm). All measures were taken to the nearest millimetre, using set points on the body (bony landmarks where applicable) to allow their use on a variety of breeds with divergent morphologies. For this study, an additional parameter, palpebral fissure width was measured. Palpebral fissure width has previously been measured via a method designed for use in unconscious (anaesthetised) animals whereby modified sliding callipers were inserted into the ventral conjunctival sac, and opened to stretch the lids maximally [28]. As this was unlikely to be tolerated in the conscious animal, and could result in distress and accidental damage to the conjunctiva and cornea, the unstretched palpebral fissure width (mm) was instead measured in the conscious animal (Fig 2) using a soft tape measure pulled taut from the medial to lateral canthus. This was held directly (<1cm) in front of the open eye, with the dog’s head gently restrained to avoid contact to the cornea. This non-invasive measurement would also be more practical for use by dog breeders and judges if it were used to assess the acceptability of an individual dog’s eye exposure. The capacity for error in the palpebral width measurements made in this study may be higher than those collected by Stades *et al* (1992), as the conscious dog is liable to move and blink [28]. In cases where movement initially disrupted this measurement, dogs were gently restrained by a veterinary nurse, and the measurement repeated three times, with a mean of these three measures recorded. Measurement of the palpebral fissure and all other measurements were performed by a single rater (RMAP) to avoid issues of inter-rater reliability. Weight (kg) was measured in all dogs on regularly calibrated digital scales, and body condition score (BCS) was assessed on the Purina 9 point scale [29] by a single rater (RMAP).

**Fig 3:**
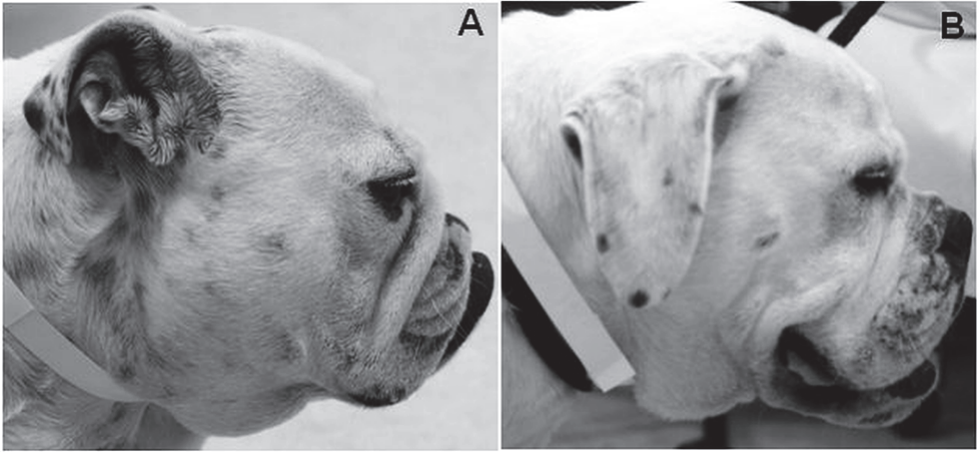
Example of dogs with and without nasal folds. A Bulldog with a marked nasal fold obscuring the nasal planum (A), and a relatively longer muzzled American Bulldog lacking this feature (B).

A further morphometric predictor of interest for corneal ulcers was craniofacial ratio, (CFR): the muzzle length divided by the cranial length, which quantifies the degree of brachycephaly, was used to differentiate skull morphologies [30]. All dogs were examined for the presence of a nasal fold; defined as a discernible fold of skin on the dorsal surface of the muzzle that was present without manipulation of the skin, and could be easily grasped between vernier callipers. This was recorded as a binary trait, and whether the fold was ‘unbroken’ or ‘broken’ i.e. extended over the muzzle as one continuous fold or not. Fig 3 demonstrates a dog with a marked nasal fold which obscures the nasal planum when viewed in profile, and in contrast, a similar brachycephalic dog with a relatively longer muzzle, and no discernible fold.

All dogs were examined for the visibility of the white part of their eyes (sclera) when looking directly forwards. This was carried out by gaining the dog’s attention (using a toy or treat, for example) and taking a photograph using a digital camera (Canon EOS 500D, Taiwan) for analysis and confirmation. Dogs were not restrained for this activity, to avoid manipulation of facial skin which could result in artificially exposing areas of sclera. The overall presence of visible sclera was recorded as a binary trait, and further broken down into whether this was visible medially, laterally, dorsally or ventrally, and a score of 0–4 was designated for each dog dependent on how many quadrants of sclera were visible.

#### Statistical analysis

Data were analysed using generalised linear mixed models (GLMM) for binary outcomes in R, using lmer from the lme4 package. The presence (or history) of corneal ulcers was the binary response variable. The morphometric variables of interest were palpebral fissure width, relative muzzle length (craniofacial ratio), and the presence of a nasal fold. Principal component analysis (PCA) of the remaining breed-defining measurements was carried out using IBM SPSS v21, to extract principal component 1 (PC1), to attempt to replicate a variable previously documented to demonstrate overall skeletal size [31]. Muzzle length, cranial length and palpebral fissure width were omitted from the PCA, so these variables were not included in the statistical models twice. PC1 explained a total variance of 77%, and factor loadings for all variables were positive.

To investigate the effect of palpebral fissure width, it was tested in models as both an absolute and a relative measure. To make this measurement relative to the overall skull size, and thus comparable between dogs of different sizes, it was divided by cranial length. Relative palpebral fissure width = (Palpebral fissure width (mm) / (Cranial length (cm) x 10)) x 100.

Multicollinearity was experienced between the morphometric measures of interest, with relatively short muzzles associated with the presence of a nasal fold, larger relative palpebral fissure width, and increased scleral exposure. To overcome this problem, and simultaneously examine the relative effects of these three variables, the craniofacial spectrum was divided into two categories; to represent brachycephalic dogs (craniofacial ratio <0.5; i.e. muzzle less than 50% the length of the cranium) and non-brachycephalic dogs (craniofacial ratio >0.5). To examine the effect of craniofacial ratio as a continuous factor in isolation, a univariable model was further constructed. The relationship between scleral exposure (as a binary trait) and ulcer risk was also examined using a univariable model, and the relationship between degree of sclera exposure (as an ordinal scale of 0–4 areas exposed) with relative palpebral fissure width was investigated using a Spearman’s rank test.

In all models breed was included as a random effect, coded using Kennel Club breeds, and all cross breeds coded plainly as ‘cross breed’ due to the unknown parentage of many of these dogs. This random effect took into account the genetic non-independence of multiple members of the same breed in the study population, and possible demographic and environmental factors that may alter risk of trauma to the cornea, such as owners of some breeds being more likely to live in certain areas (e.g. rural *vs* urban), or housing types (e.g. apartments *vs* houses). Non-morphometric predictors tested as fixed effects e.g. continuous factors such as age (years), and categorical factors such as sex (male/female), neuter status (yes/no), BCS (1–9) and genetic/cultural grouping (Parker breed groups and Kennel Club groups) were considered in all models. Multicollinearity was checked for in all models (identified from inflated standard errors in the models). Model fit was assessed using the deviance and Akaike's information criterion. From the model output, equations were used to calculate the probability of being affected by corneal ulcers at different values of relative palpebral fissure width, varying whether the dog was brachycephalic and whether it had a nasal fold to demonstrate the effects of these risk factors.

## Results

### Demographics—overall population

A total of 700 dogs were included in the study population. Of the overall population, 13% were cross breeds and 87% pure bred, with 97 breeds represented. The five most common breeds were the Labrador Retriever (56 dogs, 8%), German Shepherd Dog (36 dogs, 5.1%), Dachshund, Miniature Smooth Haired (32 dogs, 4.6%), Pug (32 dogs, 4.6%), and Border Collie (28, 4%). Three hundred dogs were female (43%) *versus* 400 males (57%) with the majority (72%) neutered. The mean±SE weight (kg) was 21.5± 0.55. The median BCS was 5 (range: 2–8) with about half (51%) an ideal BCS of 4–5. Only 21 dogs were considered underweight (3%), in contrast to 328 overweight dogs (46%) with a BCS of >5. The mean±SE age at diagnosis was 5.17±0.13 years (range 3 months—15 years 3 months).

### Demographics—affected dogs

A total of 31 dogs were diagnosed with corneal ulcers, either after referral for this condition (n = 6), after diagnosis of this condition while in hospital for another disorder (n = 11), or having previously been diagnosed with an ulcer and this documented in the dogs’ history (n = 14). Thirty of these cases were pure bred dogs, representing 10 different breeds (Table 3), the most common being the Pug (n = 12 affected), the Shih Tzu (n = 4), the Bulldog and the Cavalier King Charles Spaniel (n = 3). The breeds with the highest proportion of dogs affected were the Pekingese (2/3), Pug (12/32) and Shih Tzu (4/13). The majority of ulcer cases were male (71%), small dogs (mean weight 11.4kg ± 1.1), with a mean age of 2.8 years ±0.46.

**Table 3:** Breeds and relevant conformations of dogs affected by corneal ulcers.

Breed	Mean relative palpebral fissure width ± SE	Mean craniofacial ratio ± SE	Number of cases (n)	Percent of corneal ulcer cases (%)	Total breed population	Percent of breed affected (%)
Overall population mean	22.1 ± 0.16	0.51 ± 0.01				
Pekingese	34.18 ± 0.53	0.12 ± 0.01	2	6.5	3	66.7%
Pug	30.06 ± 0.78	0.08 ± 0.01	12	38.7	32	37.5%
Shih Tzu	28.53 ± 0.59	0.20 ± 0.01	4	12.9	13	30.8%
Bulldog	20.70 ± 0.53	0.22 ± 0.15	3	9.7	16	18.8%
Boston Terrier	26.78 ± 1.13	0.15 ± 0.01	1	3.2	6	16.7%
Pomeranian	28.77 ± 0.84	0.43 ± 0.04	1	3.2	6	16.7%
French Bulldog	23.59 ± 0.85	0.19 ± 0.13	2	6.5	13	15.4%
Cavalier King Charles Spaniel	26.99 ± 0.51	0.40 ± 0.01	3	9.7	26	11.5%
Staffordshire Bull Terrier	22.90 ± 0.85	0.51 ± 0.02	1	3.2	16	6.3%
Labrador Retriever	18.97 ± 0.31	0.58 ± 0.01	1	3.2	56	1.8%
Cross Breed	22.38 ± 0.38	0.54 ± 0.01	1	3.2	91	1.1%

Prevalences are also shown by breed.

### Modelling the risk of corneal ulcers

All four morphometric factors were significant in the final GLMM analysis: relative palpebral fissure width, the presence of a nasal fold, the brachycephalic skull morphology, and exposed sclera (Table 4). Dogs with nasal folds were nearly five times more likely to be affected by corneal ulcers than those without, with the majority of affected dogs having a nasal fold (65%), in comparison with a minority of unaffected dogs (7%). Brachycephalic dogs (craniofacial ratio <0.5) were twenty times more likely to be affected than non-brachycephalic dogs. No effects of overall size (PC1), BCS, weight, genetic/cultural grouping or signalment were found in any models.

**Table 4:** Results of binary response mixed model analysis of morphometric predictors upon corneal ulcer risk.

Predictor	Sub category	OR (95% CI OR)	SE	Z	Sig
**Nasal fold**	Yes	4.84 (2.05–11.4)	0.44	3.60	<0.001
	No	(ref)			
**Relative palpebral fissure width (%)**	-	1.12 (1.03–1.22)	0.04	2.78	0.005
**Brachycephalic (craniofacial ratio <0.5)**	Yes	20.03 (2.48–161.6)	1.07	2.81	0.005
	No	(ref)			

The response variable was presence or absence of corneal ulceration. ‘(ref)’ indicates the reference category; ‘OR’ refers to the odds ratio and SE the standard error of the OR.

Absolute palpebral fissure width was non-significant in all models; however, it was highly significant when included as a measure relative to skull length. The mean ± SE relative palpebral fissure width was higher for affected dogs (27.7% ± 0.90) than unaffected dogs (21.8% ± 0.15), i.e. affected dogs had wider eyelid apertures relative to their skull lengths than unaffected dogs. A 1% increase in relative palpebral fissure width was found to increase the risk of ulcers by 1.12. As an illustrative example, the mean relative palpebral fissure width for a Labrador Retriever was 19.0 and for a Pekingese was 34.2, a difference of over 15%. This 15% increase equates to an increased odds of 5.47.

**Fig 4:**
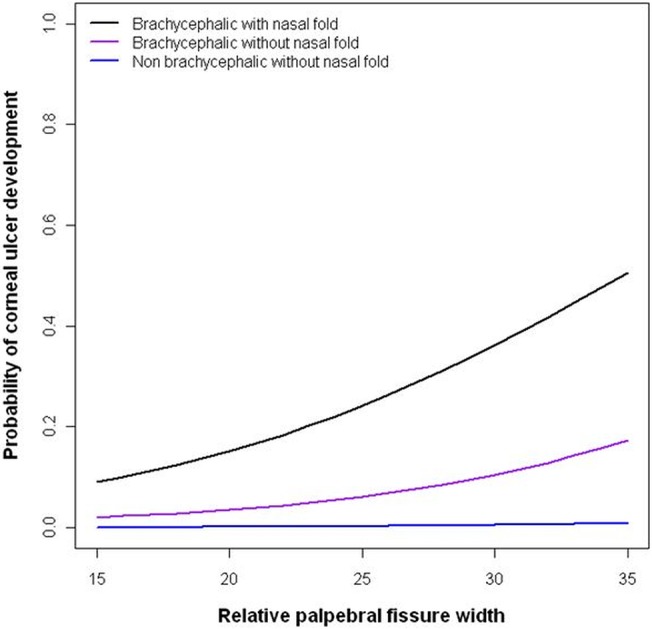
Probability of being affected by a corneal ulcer across the relative palpebral fissure width spectrum. The curves are generated from a generalised linear mixed model that included the presence of a nasal fold, relative palpebral fissure width and craniofacial conformation as predictors for corneal ulceration, and breed as a random effect. Brachycephaly, the presence of a nasal fold, and a relatively wider palpebral fissure all significantly increased the risk of ulceration. Brachycephalic dogs with and without nasal folds, and non brachycephalic dogs without nasal folds are represented, with brachycephalic dogs with skin folds at highest risk throughout the spectrum.

Breeds with the highest mean relative palpebral fissure width values were predominantly small, brachycephalic breeds, with the three most extreme breeds being the Pekingese, Griffon Bruxellois and Pug; the only breeds with mean values over 30% (Table 3). In addition to the affected breeds listed in Table 3, three further breeds should be considered to have high-risk morphologies due to their high relative palpebral fissure width values; the Griffon Bruxellois (mean±SE relative palpebral fissure width: 31.24 ± 0.47), Lhasa Apso (27.93 ± 1.73) and Boxer (26.77 ± 0.88). The contributing effects of a relatively large palpebral fissure, a brachycephalic morphology and a nasal fold on the risk of ulceration is demonstrated in Fig 4.

**Fig 5:**
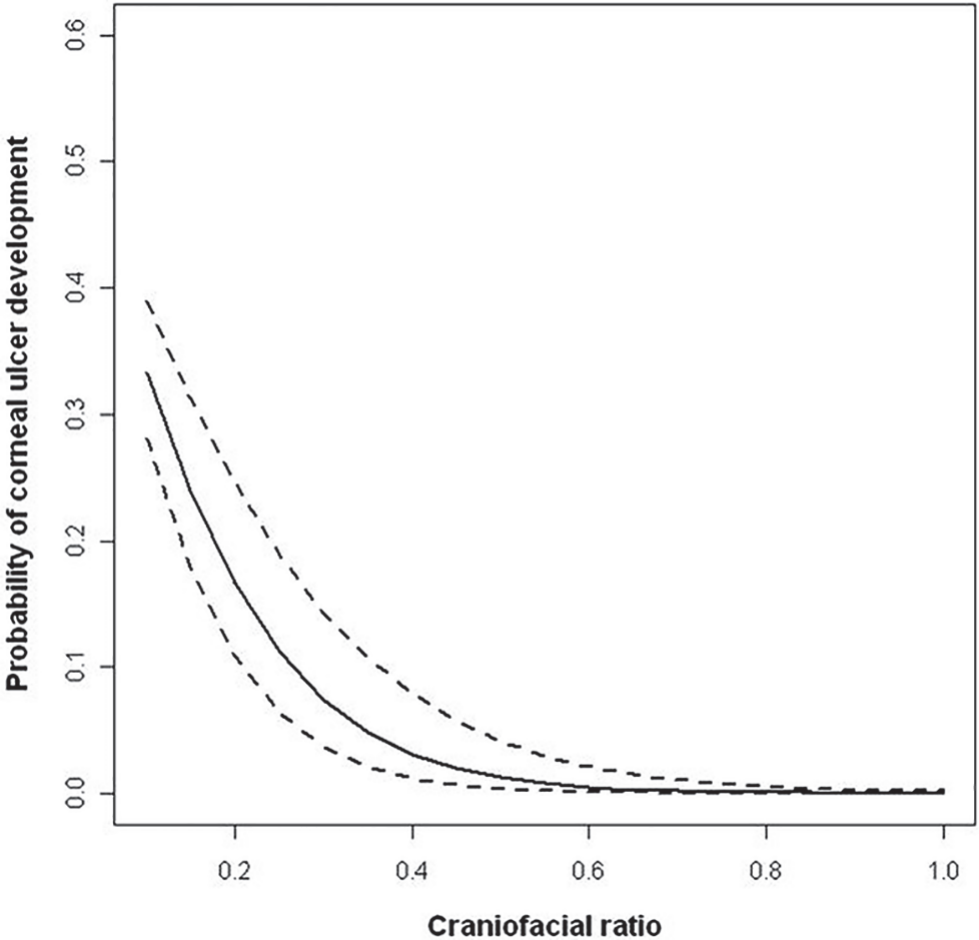
Probability of being affected by a corneal ulcer across the craniofacial ratio spectrum. The curve is generated from a generalised linear mixed model with craniofacial ratio as a single continuous factor to examine its effect on ulcer risk in isolation. As craniofacial ratio increases (and muzzles become relatively longer), corneal ulcer risk decreases.

The mean craniofacial ratio of affected dogs was shorter than unaffected dogs (0.20 ± 0.03 vs. 0.53 ± 0.01); with these mean ratios describing moderately brachycephalic and non-brachycephalic morphologies, respectively. When craniofacial ratio was included as a continuous variable in a univariate analysis, increasing muzzle length was significantly associated with decreasing ulcer risk (OR ± SE: -9.14 ± 1.25 (95% CI: -11.5 –-6.70); p<0.001) (Fig 5).

Visible sclera was present in 35.3% of all 700 dogs, with the most common location being ventrally to the iris (29.6% of dogs). The majority of dogs showed sclera in only one segment of the eye (17.1%); however in 6% it was visible in two areas, 11% in three areas, and 1% (9 dogs) in all four areas. Increased exposure of the sclera was associated with an increase in the number of dogs affected by corneal ulcers: ulceration was observed in 0%, 16%, 24% and 33% of dogs with one to four quadrants of exposed sclera, respectively. Of the 31 ulcer cases, 28 (90.3%) exhibited visible sclera, and when this was included as a binary variable in a univariate analysis, it was found to significantly increase ulcer risk (OR ± SE: 2.66± 0.08 (95% CI: 1.09–6.50); p<0.05). Dogs with four areas of visible sclera exhibited the highest relative palpebral fissure width values (mean ± SE: 29.1± 1.43) in comparison with all other groups, with dogs showing only one visible area exhibiting the lowest (mean ± SE: 20.8±0.36). Spearman’s rank tests identified a significant positive correlation between relative palpebral fissure width and number of areas of visible sclera (r = 0.31, p<0.001).

## Discussion

### Conformational risk factors for corneal ulcers

This study has confirmed four major conformational risk factors previously suspected in the development of corneal ulcers in domestic dogs; the presence of a nasal fold, the width of the palpebral fissure relative to the length of the cranium, brachycephalic skull morphology, and scleral exposure. These conformational traits are interlinked, with brachycephalic dogs more likely to have a nasal fold, having relatively larger palpebral fissures, and having more of the white part of their eye exposed. Even so, nasal folds can occur independently of skull morphology, such as in brachycephalic Boston Terriers and Affenpinschers, which rarely have nasal folds, *versus* mesocephalic Shar Peis and Chow Chows, which can possess them due to their wrinkled skin. These are exceptions to the general association between the traits, so breeding strategies to reduce the risk of corneal ulcers are likely to have to involve selecting for relatively longer muzzles. Although a craniofacial ratio of >0.5 may not be feasible for some affected breeds with substantially lower craniofacial ratios (e.g. the Pug, mean±SE: 0.08±0.01), encouraging selection for relatively longer muzzles would help reduce risk. The affected breeds in the present study were consistent with previous studies [32], but with the addition of the Pug, currently the 9^th^ most popular breed in the UK [33].

It is perhaps not surprising that the presence of a nasal fold substantially increased the risk of corneal ulcers, as nasal fold trichiasis has long been suspected cause of corneal damage [20]. To reduce the risk of corneal ulcers, breeding dogs without this facial feature would be desirable. As nasal folds are described in several breed standards and considered a desirable feature, such a change may be contested. Moreover, if they are present in a large proportion of a breed, selecting only dogs without this feature for breeding may be logistically difficult and dramatically reduce genetic diversity. As such, it may be more feasible to initially breed for less pronounced nasal folds, carefully selecting for those that are positioned more rostrally to avoid contact with the cornea. This may be more difficult in brachycephalic breeds with moderate to long hair coats (e.g. Pekingese, Shih Tzu) where hairs may still come into contact with the cornea.

The finding of a relatively larger palpebral fissure being a risk factor for corneal ulcers is novel; it has not previously been measured in a large sample of varied breeds and investigated in this way, despite years of veterinary supposition, and indeed surgical intervention to alter this feature. In a previous study of risk factors for corneal ulcers it was noted that lagophthalmos in brachycephalic breeds must be treated properly to prevent ulcers [32]; however in the longer term we can go one step further than this, by breeding for dogs with morphologies that do not necessitate surgical intervention to return them to a lower risk morphology. As asserted by Bedford (1998), if the characteristic appearance of a breed, as dictated by breed standards, is associated with clinical problems, then redefinition of such standards are required if these associated problems are to be eliminated. Positive selection towards relatively smaller eyelid apertures should be encouraged [34]. For example, the mean relative palpebral fissure width of Pugs in this study was 30%, which corresponds to a 0.36 probability of being affected by corneal ulcers if accompanied by a nasal fold; if the relative width were to be reduced to 20%, then the probability as calculated by the model here would markedly decrease to 0.15. The measurement and calculation of relative palpebral fissure width outlined here is simple and could be carried out by breeders at home. This may be a useful way to objectively differentiate between their dogs’ morphologies when making breeding decisions. Fig 2 demonstrates how to take the measurements of interest, and then calculate craniofacial ratio and relative palpebral fissure width. That higher relative palpebral fissure width values are associated with increased scleral exposure will also be of practical use, by highlighting dogs with the highest relative palpebral fissure width values in an easily discernible manner. This may be particularly useful in situations where identifying the most at-risk dogs is important, but measurement is not feasible, for example by judges in the show-ring.

Turning to the third factor, the brachycephalic morphology was the risk factor identified as having the greatest effect upon risk of ulcer development. This is likely to be due to both direct consequences of this morphology, but also its association with other risk factors for ulcers not studied here. Directly, foreshortened muzzles are associated with shallow orbits, resulting in exophthalmic eyes [17] that are subsequently at risk of traumatic ulceration due to their degree of protrusion. In other words, protruding eyes that are not protected within a sufficiently deep orbit are susceptible to collision with, or abrasion against, external objects. Also, they cannot fully blink, leading to areas of chronic drying and consequent corneal damage. In a recent study of ulcerative keratitis (32 cases), the majority of cases were brachycephalic, with 50% represented by the Shih Tzu and 25% by the Pekingese [32]. The most frequent aetiology of ulcerative keratitis in brachycephalic breeds was found to be corneal exposure due to lagophthalmos, whereas it was keratoconjunctivitis sicca in non-brachycephalic breeds [32].

Some brachycephalic breeds are also affected by an array of ophthalmic abnormalities further predisposing to ulcers, including lower medial entropion, an inward rotation of the eyelid, often obscured by the nasal fold [18], and distichiasis, where abnormally growing eyelashes come into contact with the cornea [35]. Corneal sensitivity or corneal touch threshold, the minimum stimulation of the corneal surface required to elicit a blink reflex, is also relatively lower in brachycephalic dogs [36]. This makes them less responsive to irritants that may cause damage to the surface of the eye, e.g. which otherwise would be washed away by blinking and/or tear production [6]. The decreased sensitivity may also lead to an increased risk of traumatic injury, and allow ulcers in the early stages to go unnoticed by owners due to a lack of overt behavioural signs of ocular discomfort and pain.

Brachycephalic dogs are additionally predisposed to tear film abnormalities that are thought to increase ulcer risk. Brachycephalic dogs often have a thin lipid layer in the tear film and decreased aqueous coverage in the central cornea as a result of their incomplete blinking [37]. Several brachycephalic breeds have been identified as being predisposed to dry eye, including the Bulldog, Lhasa Apso, Shih Tzu, Pug, Pekingese, Boston Terrier and Cavalier King Charles Spaniel [38]. Even moderately lowered tear production associated with dry eye may produce clinical signs in brachycephalic dogs, as a larger portion of the globe is exposed [39]. In a UK based study, a higher proportion of brachycephalic dogs that were affected by dry eye were also affected by ulcers, than were non-brachycephalic dogs with dry eye, e.g. 36% of Shih Tzus and 30% of Cavalier King Charles Spaniels *versus* 17% of dogs in the overall study population [40]. In the current study, no attempts were made to assess the presence of dry eye as a risk factor for corneal ulcers; however, any breeding programme aimed at reducing ulcer prevalence should include dry eye as a disorder to avoid in breeding dogs. Due to the potential influence of dry eye on corneal ulcer risk, future studies focussing on eye abnormalities not investigated here, such as lacrimal disorders (using both quantitative and qualitative evaluation), along with other common abnormalities in brachycephalic dogs such as conformational disorders of the eyelids (e.g. entropion) may shed further light on the variability of ulcer risk between dogs.

### Ways forward

**Fig 6:**
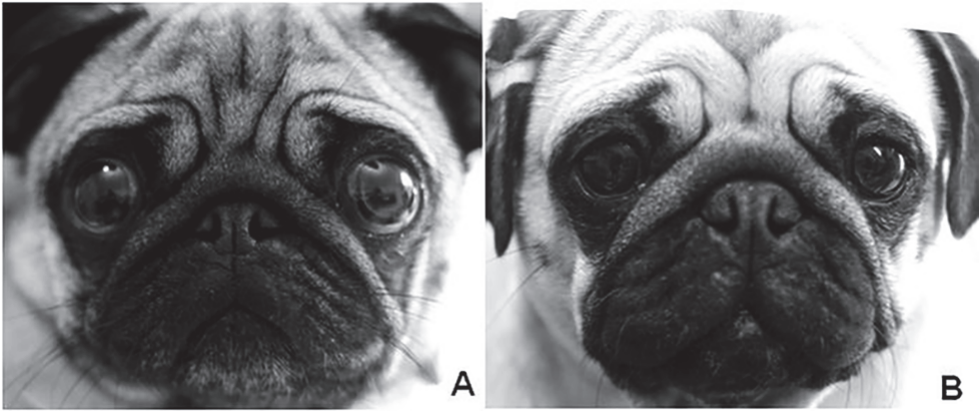
Differentiating between higher and lower risk eye morphologies. The Pug in (A) exhibits an extremely high-risk conformation for corneal ulcers, with a relative palpebral fissure width of 35%, compared to the Pug in (B), who exhibits a lower relative palpebral fissure width of 30% and correspondingly lower risk. It should be noted that 30% is still a high risk morphology, and should not be aimed for in most breeding programmes. The amount of visible sclera is easily discernible between these two dogs. Both dogs have a prominent nasal fold and are extremely brachycephalic.

With canine welfare in mind, it is recommended that breed standards are further amended to discourage these traits, promoting low-risk morphologies. This recommendation has already been supported by many stakeholders in a recent conference on brachycephalic health; for example, 83% of stakeholders voted that exposed sclera (the white part of their eye) should *never* be described in breed standards, 42% voted that ‘large’ should never be used to describe eye size, 59% voted that nasal folds should never be described, and 43% voted that the word ‘short’ should not be used to describe muzzle length in breed standards [41]. At the same conference, The Kennel Club and veterinarians were voted two of the stakeholder groups most responsible in safeguarding the future health and welfare of brachycephalic dogs. In conjunction with other organisations with the available resources and links with the public (e.g. animal welfare charities), these groups have a responsibility in educating the dog owning public, breeders and potential puppy buyers regarding conformational risk is of importance in any attempts to reduce the prevalence of corneal ulcers. Encouraging puppy buyers to select more moderate dogs even within high-risk breeds i.e. those with a sire and dam with less prominent eyes, longer muzzles and reduced or absent nasal folds would be a positive step in creating demand for a healthier phenotype that is at lower risk of developing ulcers. The use of images to illustrate dogs of different conformations may be a useful tool. For example Fig 6, illustrates the difference between a very high risk and a slightly lower risk (but still relatively high risk) Pug, with obvious differences in the amount of exposed sclera.

Educating dog owners that the conformation of their dog can and does affect corneal ulcer development is of importance. It has been noted that following palpebral fissure shortening, despite the smaller appearance of the eye after surgery, most owners were satisfied with the results; however, the same authors also noted that owners may be reluctant to have a nasal fold resection performed, despite it being indicated by their veterinary surgeon [22]. It was not stated whether this was due to aesthetics, or other concerns; however, if the former, the relative moral importance of health over appearance must be made clear to the client.

## Conclusions

This study supports the notions that the brachycephalic skull shape, relatively large eyelid openings, nasal folds and exposed sclera, often encouraged by breed standards, are risk factors for the development of corneal ulcers. Breeders and buyers of affected breeds should select dogs with more moderate eye sizes, relatively longer muzzles, and less pronounced or preferably no nasal folds, to reduce the risk of this painful condition and avoid the necessity of surgical treatment in their dogs. It is acknowledged that the aetiology of corneal ulcers is often multi-factorial and complex, with variability even between dogs of the same breed; however, this only serves to highlight the need for a multi-faceted action plan to reduce the prevalence of corneal ulcers in severely affected breeds. In summary, to reduce ulcer risk, the results presented here suggest that dogs exhibiting any of the four high risk conformations—especially in combination—should be avoided in breeding programmes, should not be awarded in the show-ring by judges, and should not be encouraged in breed standards.

## Supporting Information

S1 Table Section of owner questionnaire regarding eye health. (DOCX)

S1 Dataset Raw data from study. (XLSX)
