# Supplementary material for: Impact of Facial Conformation on Canine Health: Corneal Ulceration
Source: PLoS One. 2015 May 13;10(5):e0123827. doi: 10.1371/journal.pone.0123827 (PMC4430292; doi:10.1371/journal.pone.0123827)
Supplement: S1 Table — (DOCX) [file pone.0123827.s001.docx]

Supplementary Table 1.Section of owner questionnaire regarding eye health

| **Does your dog currently have, or have**  **a history of EYE PROBLEMS?** | | | | | **YES** | | | | | **NO** | | |
| --- | --- | --- | --- | --- | --- | --- | --- | --- | --- | --- | --- | --- |
| **IF YES, PLEASE ANSWER THE FOLLOWING QUESTIONS:** | | | | | | | | | | | | |
| - If known, what was your dog diagnosed with? | | |  | | | | | | | | | |
| - What treatment (IF ANY) has your dog received for this condition?   (PLEASE TICK AND FILL IN ANY ADDITIONAL INFORMATION) | | |  | Surgery | | | | |  | | | |
|  |  |  |  | Medication | | | | |  |  |  |  |
| At what age did you first notice this condition? | | |  | | | | Years | |  | | | Months |
| What first made you notice this condition? e.g. change in the appearance of the eye, change in dogs’ behaviour (PLEASE STATE) | | |  | | | | | | | | | |
| - How quickly did these signs appear? - (PLEASE CIRCLE) | Suddenly over a few hours | | Over a few days | | | Gradually over a few weeks | | Gradually over several months | | | Gradually over longer than 1 year | |
| Do you believe this condition is:  (PLEASE CIRCLE) | Resolved | Getting better | Getting worse | | | Staying the same | | Comes and goes (but always there) | | | Episodic- sometimes free of problem | |
| If episodic, how many episodes have occurred: |  | | | | | | | | | | | |
| If your dog has previously been treated for this condition, for how long were they ‘improved’? |  | | | | | | | | | | | |
